# Correlation between ApoE gene polymorphisms and the occurrence of urolithiasis

**DOI:** 10.3892/etm.2014.2047

**Published:** 2014-11-04

**Authors:** BIAO QIAN, LIYING ZHENG, QINZHANG WANG, GUOFU DING

**Affiliations:** 1 Department of Urology, The First Affiliated Hospital of Shihezi University School of Medicine, Shihezi, Xinjiang 832003, P.R. China; 2 The Office of In-Hospital Infection Control, The First Affiliated Hospital of Shihezi University School of Medicine, Shihezi, Xinjiang 832003, P.R. China

**Keywords:** urolithiasis, dyslipidemia, apolipoprotein E gene polymorphism, Uyghur population

## Abstract

The aim of the present study was to investigate the correlation between apolipoprotein E (ApoE) gene polymorphisms and the occurrence of urolithiasis and dyslipidemia. A total of 180 Uyghur individuals, including 90 urolithiasis patients and 90 healthy controls, were enrolled in this study. The blood lipid profiles of the patients and controls were investigated and compared, and the composition of the urinary calculi was determined. The polymorphisms of the ApoE alleles were analyzed using polymerase chain reaction-restriction fragment length polymorphism analysis. Three common genotypes of the ApoE gene, E3/3, E3/4 and E4/4, were detected in the urolithiasis patients and control group. In the patient group, 28 patients with the E3/3 genotype (30.1%), 58 patients with the E3/4 genotype (64.4%) and four patients with the E4/4 genotype (4.5%) were identified. By contrast, in the control group, 52 patients with the E3/3 genotype (57.8%), 35 patients with the E3/4 genotype (38.9%) and three patients with the E4/4 genotype (3.3%) were identified. The frequency of the E3/4 genotype was found to be significantly higher in the patient group when compared with the control group (χ^2^=12.96; P<0.001). In addition, the frequency of the E4 allele was significantly higher in the patient group when compared with the control group (χ^2^= 6.61; P=0.025). In conclusion, the occurrence of urolithiasis was found to be associated with ApoE gene polymorphisms, and the E4 allele may be a potential susceptibility factor for urolithiasis.

## Introduction

Urolithiasis is a common urinary disease, with increasing incidence worldwide. The occurrence of urolithiasis varies among geographical regions and ethnic groups. A high incidence of urolithiasis has been observed in Xinjiang, China, particularly among the Uyghur population of south Xinjiang that follows a high-fat diet. High levels of blood lipids have been reported to affect lipid metabolism and may be a risk factor for urolithiasis (1,2). Blood lipid levels are affected by genetic factors and lifestyle, and lipid metabolism processes are regulated by a set of genes. Different blood lipid levels have been detected among ethnic groups as early as childhood, which indicates that the differences may be based on genetic factors (3). In addition, genetic factors have been identified as a possible cause of a number of abnormalities in blood lipid metabolism (4). The present study focused on the apolipoprotein E (ApoE) gene, which is one of the components of plasma lipoproteins. ApoE regulates blood lipid metabolism by binding with a receptor protein (5). Epidemiological investigations have revealed that ApoE gene polymorphisms are closely associated with lipid metabolism abnormalities (6).

ApoE is a major plasma lipoprotein that plays an important role in lipoprotein metabolism. Lipoproteins with different ApoE isomers have been reported to exhibit distinct kinetic characteristics and affect the blood lipid levels differently (7). Kong *et al* (8) reported that cholesterol crystallization in the gall bladder was associated with ApoE polymorphism, and hypothesized that ApoE may be a promoter of nucleation, and thus, a susceptibility factor for cholesterol crystallization in the gall bladder. In the present study, lipid metabolism was investigated in urolithiasis patients among the Uyghur population. Using polymerase chain reaction-restriction fragment length polymorphism (PCR-RFLP) analysis, the associations between ApoE gene polymorphisms and lipid metabolism abnormalities were analyzed.

## Materials and methods

### Subjects

In total, 90 Uyghur patients with urolithiasis from the Aksu Prefecture, hospitalized between January and July 2007, were enrolled in this study (male, 51; female, 39; age range, 7–67 years). In addition, 90 healthy Uyghur individuals with no blood relation to the patients were randomly selected as the control group (male, 51; female, 39; age range, 8–69 years). Individuals from the control group had similar occupations and resided in the same area as the patient group individuals. B-type ultrasonography was performed to ensure that the control group did not suffer from a urinary calculus or any other relevant diseases. Patients diagnosed with a urinary calculus who did not undergo removal of the calculus, even following extracorporeal shock wave lithotripsy, as well as patients with chronic urinary infections and renal insufficiency, were excluded from the study. Qualitative analysis of the calculus components was conducted using standard calculus qualitative analytical chemical reagents supplied by the Institute of Urology, Peking University (Peking, China). The reagents included calcium phosphate, calcium oxalate, ammonium magnesium phosphate, uric acid, carbapatite, and cystine. The stone samples were powdered and analyzed by Fourier transform infrared spectrophotometry (Tensor 27; Bruker Optics GmbH, Ettlingen, Germany).

Prior written and informed consent was obtained from all the patients and the study was approved by the Ethics Review Board of Shihezi University (Shihezi, China).

### Measurement of blood lipid profiles

Blood lipids levels were analyzed on an Olympus AU400 Automated Chemistry analyzer (Olympus Optical Co., Ltd., Tokyo, Japan)*.* Blood samples were collected from the peripheral vein of each individual without fasting and frozen in liquid nitrogen. Aliquots of frozen serum were thawed on ice for 2 h. The samples were thus frozen and thawed twice in total. Lipid metabolites were extracted from 100 μl of serum. Blood levels of cholesterol and triglycerides were analyzed in the patients and control groups using the cholesterol oxidase method. In addition, the levels of apolipoprotein A-I and total lipoprotein were determined by an immunoturbidimetric assay. The levels of high-density lipoprotein and low-density lipoprotein were measured using a routine Hitachi 7600 autoanalyzer (Hitachi High-Technologies Corporation, Tokyo, Japan).

### PCR-RFLP analysis

A 5-ml blood sample was collected from the peripheral vein of each individual and was anticoagulated with EDTA. DNA was extracted from the blood sample using a Genomic DNA Extraction kit (Sangon Biotech Co., Ltd., Shanghai, China), according to the manufacturer’s instructions. The ApoE gene was amplified in a total volume of 30 μl. The primerswere synthesized by Sangon Biotech Co., Ltd. and their sequences as follows: forward: 5′-ACA GAA TTC GCC CCG GCC TGG TAC AC-3′ and reverse: 5′-TAA GCT TGG CAC GGC TGT CCA AGG A-3′. Each PCR cycle included 3 μl 10X PCR buffer (with 15 mM MgCl_2_), 2 units *Taq* DNA polymerase (Takara Bio, Inc., Tokyo, Japan), 2 μl dNTP (2.5 mM), 0.4 μl each of the forward and reverse primers (20 mM) and 0.5–0.6 μg DNA templates. The following PCR procedure was used: initial denaturation at 97°C for 7 min; 35 cycles of denaturation at 95°C for 45 sec; annealing at 65°C for 45 sec and extension at 72°C for 1 min; and a final extension at 72°C for 10 min. PCR was performed in a C1000 Touch^™^ PCR thermal cycler (Bio-Rad, Hercules, California, USA)

Following PCR, a 1-μg sample of the ApoE gene amplification product was digested with 20 μl restriction endonuclease (Sangon Biotech Co., Ltd.). Following digestion, the DNA fragments were separated by electrophoresis on a polyacrylamide gel for 2 h at 90 V. The genotypes were identified using a Gel Doc^™^ XR gel imaging and analysis system (Bio-Rad). A sample was randomly selected from each genotype identified during the RFLP analysis, and sequenced by Sangon Biotech Co., Ltd.

### Statistical analysis

The frequencies of the genotypes and gene alleles were calculated separately in the patient and control groups using a gene counting method as described previously (9). The data were tested for Hardy-Weinberg equilibrium and statistical analysis was performed using SPSS 17.0 software (SPSS, Inc., Chicago, IL, USA). The genotypic and allelic differences between the control and patient groups were examined with the χ^2^ test, where P<0.05 was considered to indicate a statistically significant difference.

## Results

### Analysis of calculus composition

To determine the percentage of each component in the calculi of the patients, calculus composition was analyzed using standard calculus qualitative analytical chemical reagents. The results indicated that the major component of the calculi was calcium oxalate in 90.0% of the patients (81/90). Among the 81 patients with calcium oxalate calculi, the calculus composition detected was as follows: Pure calcium oxalate (67.9%, 55/81); calcium oxalate and calcium phosphate (4.9%, 4/81); calcium oxalate and ammonium magnesium phosphate (2.5%, 2/81); calcium oxalate and uric acid or ammonium urate (18.5%, 15/81); calcium oxalate, ammonium magnesium phosphate and dahllite (2.5%; 2/81); calcium oxalate and dahllite (2.5%, 2/81); and calcium oxalate and cystine (1.2%, 1/81). Among the other nine urolithiasis cases, the calculus composition included pure calcium phosphate (2.2%, 2/90), pure uric acid (5.6%, 5/90) and pure ammonium magnesium phosphate (2.2%, 2/90). These results indicated that the major component of calculi in urolithiasis cases among the Uyghur population of south Xinjiang was calcium oxalate.

### Number of cases with abnormal levels of total cholesterol, triglycerides and high-density lipoproteins is higher in urolithiasis patients

Blood lipid levels were measured to investigate the role of blood lipids in urolithiasis. The blood lipids detected included total cholesterol, triglycerides, high-density lipoproteins, low-density lipoproteins, apolipoprotein A-I and lipoproteins. As shown in Table I, abnormal levels of total cholesterol were detected in 38 urolithiasis patients (42.2%) and 21 individuals from the control group (23.3%). In addition, abnormal levels of triglycerides were observed in 33 patients (36.7%) and 15 cases from the control group (16.7%). In total, 29 cases (32.2%) in the patient group exhibited abnormal levels of high-density lipoproteins, whereas abnormal levels were detected in only 16 cases from the control group (17.8%). Abnormal levels of apolipoprotein A-I were detected in 67 cases (74.4%) in the patient group and 60 cases (66.7%) in the control group. The number of cases exhibiting abnormal levels of low-density lipoproteins was 57 in the patient group (63.3%) and 53 in the control group (58.9%). Only one case (1.1%) was detected with abnormal levels of lipoproteins in the patient group, while no cases were detected in the control group. Statistically, the number of cases with abnormal levels of total cholesterol, triglycerides and high-density lipoproteins was significantly higher in the patient group when compared with the control group. However, the difference in the number of cases with abnormal levels of apolipoprotein A-I, low-density lipoproteins and lipoproteins between the two groups was not found to be statistically significant. These results indicated that dyslipidemia may be a significant factor in the development of urolithiasis among the Uyghur population of south Xinjiang.

### Associations between ApoE gene polymorphisms and the occurrence of urolithiasis

**Figure 1:**
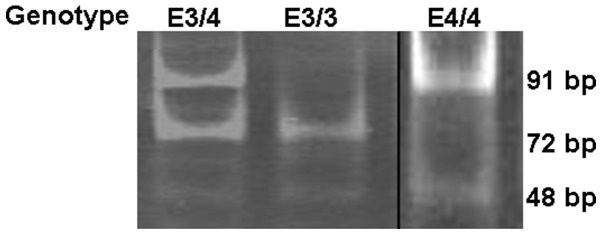
Apolipoprotein E gene polymorphisms were analyzed by polymerase chain reaction-restriction fragment length polymorphism. Following digestion with the restriction endonuclease, Hha I, the DNA fragments were run on a polyacrylamide gel and the representative electrophoresis results of the E3/4 (91, 72 and 48 bp), E3/3 (91 and 48 bp) and E4/4 (72 and 48 bp) genotypes are shown.

To determine the associations between the polymorphisms of ApoE and the occurrence of urolithiasis, ApoE gene polymorphisms were analyzed using PCR-RFLP. The ApoE gene was successfully amplified in the 180 subjects (90 patients and 90 controls) and the product size of the gene was 244 bp. In theory, due to the presence of different bases at sites 112 and 158 of the ApoE gene, three alleles and six genotypes can be generated following digestion with the Hha I restriction endonuclease. However, in this study, only three genotypes were identified in the subjects, namely, the E3/3, E4/4 and E3/4 genotypes (Fig. 1). Through Hardy-Weinberg equilibrium testing, these genotypes of ApoE were found to be consistent with the theoretical expectations.

The associations between ApoE gene polymorphisms and the occurrence of urolithiasis were analyzed. As shown in Table II, 28 cases with E3/3 (30.1%), 58 cases with E3/4 (64.4%) and four cases with the E4/4 genotype (4.5%) were detected in the patient group. By contrast, 52 individuals with E3/3 (57.8%), 35 individuals with E3/4 (38.9%) and three individuals with the E4/4 genotype (3.3%) were identified in the control group. The frequency of the E3/4 genotype in the patient group was significantly higher when compared with the control group (χ^2^=12.96; P<0.001). The number of E3 and E4 alleles in the patient group was 114 (64.8%) and 62 (35.2%), respectively, while the number of these alleles in the control group was 139 (79.0%) and 41 (21.0%), respectively. Statistically, the frequency of the E4 allele in the patient group was significantly higher when compared with the control group (χ^2^=6.61; P<0.025). The results indicated that the E3/4 genotype and E4 allele may be susceptibility factors for urolithiasis among the Uyghur population of south Xinjiang.

## Discussion

The occurrence of urolithiasis has been shown to increase with age and the increasing incidence of obesity (10). In addition to the high risk of developing obesity, coronary heart disease, dyslipidemia and abnormal glucose tolerance, individuals with metabolic syndromes also experience a high risk of urolithiasis (11). Zhang (12) reported that high levels of triglycerides, high-density lipoproteins, cholesterol and ApoE in the blood were risk factors for developing urolithiasis. Clinical control of urolithiasis may relieve the symptoms, promote the removal of calculi, lower the risk of recurrence and reduce the effect on the kidneys. Accordingly, the present study revealed that the levels of triglycerides, high-density lipoproteins and cholesterol were significantly higher in the urolithiasis patient group when compared with the control group.

A previous study demonstrated that ApoE polymorphisms were associated with human longevity in a Han Chinese population (13). In the current study, however, the association between ApoE polymorphisms and the age of the patients was not analyzed. In another study, the allele frequencies of the ApoE polymorphism in Zambian populations were 13.8, 59.5 and 26.7% for the E2, E3 and E4 alleles, respectively (14). Genotype frequencies of E3/3 were 32.8% in Zambian populations. The allele and genotype frequencies in Uyghur populations were different to those in Zambian populations. This may be due to the difference in ethnic background.

In the present study, the polymorphisms of ApoE were investigated among the Uyghur population of south Xinjiang using PCR-RFLP analysis. The number of cases with the E3/4 genotype was significantly higher in the patient group when compared with the control group (χ^2^=12.96; P<0.001). In addition, the number of cases with the E4 allele was significantly higher in the patient group when compared with the control group (χ^2^=6.61; P<0.025). Therefore, the E4 allele of the ApoE gene may be used as a potential indicator in the diagnosis and screening of urolithiasis.

## Figures and Tables

**Table I:** Blood lipid level analysis in the patient and control groups.

	Patient group, n (n=90)		Control group, n (n=90)			
				
Parameters	Normal	Abnormal	Normal	Abnormal	χ^2^	P-value
Total cholesterol	52	38	69	21	7.287	0.007^a^
Triglycerides	57	33	71	19	5.300	0.021^a^
High-density lipoproteins	61	29	74	16	5.007	0.025^a^
Low-density lipoproteins	33	57	37	53	0.374	0.541
Apolipoprotein A-I	23	67	30	60	1.310	0.252
Lipoproteins	89	1	90	0		

a P<0.05, vs. control group.

**Table II:** Associations between ApoE gene polymorphisms and the occurrence of urolithiasis.

	Genotype frequency, % (n)			Allele frequency, % (n)		
		
Group	E3/3	E3/4	E4/4	E2	E3	E4
Patient	30.1 (28)	64.4^a^ (58)	4.5 (4)	0	64.8 (114)	35.2^b^ (62)
Control	57.8 (52)	38.9 (35)	3.3 (3)	0	79.0 (139)	21.0 (41)

a P<0.001 and

b P<0.025, vs. control group.

ApoE, apolipoprotein E.
